# Research on the Driving Mechanism of Waste Separation Behavior: Based on Qualitative Analysis of Chinese Urban Residents

**DOI:** 10.3390/ijerph16101859

**Published:** 2019-05-27

**Authors:** Feiyu Chen, Hong Chen, Meifen Wu, Shanshan Li, Ruyin Long

**Affiliations:** School of Management, China University of Mining and Technology, Da Xue Road 1, Xuzhou 221116, China; chenfeiyu@cumt.edu.cn (F.C.); wumeifen6210@163.com (M.W.); shanshanli0809@163.com (S.L.)

**Keywords:** waste separation behavior, driving mechanism, qualitative analysis

## Abstract

Waste source separation is the fundamental premise to ensure effective waste recycling. Whether the entire waste recycling and reduction process can be effectively carried out depends on the waste source separation. Clarifying the driving mechanism of waste separation behavior plays an important role in effectively guiding the urban residents’ waste separation behavior and achieving waste recycling. In this study, qualitative analysis was used to explore the driving mechanism of waste separation behavior. Through the open coding, axial coding and selective coding of the in-depth interview data collected from 323 Chinese urban residents, the study has proposed and verified the four-dimensional structure of waste separation behavior, namely, waste separation behavior of habit, decision, relationship, and citizen. The main driving factors of urban residents’ waste separation behavior have been clarified. On this basis, a theoretical model for the driving mechanism of waste separation behavior was constructed in this study. Ten main categories of factors have been presented, namely, value orientation, cognition of separation, regulatory focus, preferences for comfort, perception of separation empowerment, policy and standards, products and facilities, group norms, links trustworthiness, and social demography variables. Moreover, four typical relationship structures were proposed. Finally, the intervention policy suggestions were made to effectively guide the urban residents’ waste separation behavior.

## 1. Introduction

Municipal solid waste (MSW) refers to solid waste generated in daily life or activities which provide services for daily life. It also refers to solid waste that is defined as domestic waste by laws and administrative regulations (Chen et al. 2018) [1]. It mainly comes from family houses, public places, commercial departments, public institutions, etc. (Zhang et al. 2010) [2]. Among them, the waste generated in family houses is a dominant part, accounting for about 60% of the total amount of MSW (Du et al. 2006) [3]. The total waste generated in China is large in amount and it increases year by year. In 2006, the total national waste output was 148.414 million tons. However, it increased to 203.62 million tons in 2016. In terms of per capita daily waste output, the daily per capita waste production in China in 2016 was about 1384 g, which is higher than that in 2006 (1221 g). In addition, due to factors such as population density and resource consumption, the amount of waste in large and medium-sized cities is relatively high. The *2016 Annual Report on Prevention and Control of Environmental Pollution by Solid Wastes in Large and Medium-sized Cities* issued by the Ministry of Environmental Protection of China shows that in 2015, the amount of domestic waste generated in 246 large and medium-sized cities was 18.56 million tons, accounting for 96.98% of the total national output. Therefore, the research on the separation behavior of urban residents in China is typical and representative.

MSW management is often used as an indicator to measure urban governance (Nzeadibe and Anyadike, 2012) [4]. It is even a symbol of urban health and good governance (Anantanatorn et al. 2015) [5]. As a big producer of waste, China started late in waste management and it also has many defects in the treatment of waste. Compared to developed countries like Japan and Europe, China has lagged behind in waste management (Lou, 2016) [6]. MSW is not only a major pollutant in the urban environment but it is also a resource that needs to be mined (Mühle, 2010) [7]. In order to turn waste into treasure, to achieve resource reuse and to solve the growing contradiction between supply and demand of resources and energy, reducing waste and making it harmless and resourceful is becoming more and more important (Chen and Tung, 2010) [8]. In the whole process of waste collection, residents’ separation of waste is the initial link, which is also called the source separation. The effectiveness of the source separation process not only determines the difficulty of waste collection, transportation and processing, but also affects the efficacy and trust of each link in achieving waste recycling and reduction. Therefore, waste source separation is the fundamental premise to ensure effective domestic waste recycling. It also determines whether the entire waste recycling and reduction process can be carried out effectively or not (Andrews et al. 2013) [9]. In our previous research, we found that China’s waste source separation has the drawbacks of market chaos, disorderly operation, inefficiency and secondary pollution (Chen et al. 2019) [10], which fundamentally leads to the waste of resources and frequent waste siege. Therefore, it is extremely urgent to explore the factors that hinder the waste separation of China’s urban residents, and it is of great significance to guide the waste source separation for China’s urban residents.

Most of the existing studies focus on the behavior of domestic waste management, especially the recycling behavior of domestic waste from the perspective of social psychology. Scholars explored the behavior of household waste management. They tried to identify the factors related to household waste management behavior and discuss the regularity of their behavior by studying the residents’ awareness and attitude in the domestic waste management. By summarizing the current views, the authors found that the main reasons which hinder residents’ MSW management behavior include: (1) Insufficient public awareness of environmental protection and environmental literacy; (2) Waste separation and identification system is not easy to understand; (3) Incomplete policies and regulations;(4) Relatively insufficient economic input; and (5) Slow implementation of products and infrastructure (Mee and Clewes, 2004) [11]. Existing research is of great significance in controlling, guiding and cultivating people’s domestic waste management behavior. It has also provided a sociological and psychological basis for the formulation of ecological environmental policies and the promotion of environmental protection technologies to achieve sustainable urban development. However, further research is needed in the following aspects: (1) the interaction between influencing factors. Individual behaviors are presented through the results of complex decisions. In the process of urban household waste separation, residents will make corresponding behavior decisions based on various considerations. Therefore, to understand how factors interacts with each other to drive urban household waste separation behavior and comprehensively grasp the driving mechanism of behavior is a prerequisite for effective intervention in waste separation behavior; (2) the research method needs to be enriched, especially the method to explore multiple variables and their diverse relationships. Based on the above analysis, this study intends to build a theoretical model of the driving mechanism of urban residents’ waste separation behavior through literature analysis and qualitative research, so as to provide policy recommendations for effectively guiding urban residents’ waste separation behavior. The main research stages of this research are as follows. The first part expounds the background, significance and related main concepts of this research; the second part is the literature review, combing the theoretical research related to this paper; the third part introduces the research methods used, the data collection process and the results; the fourth part is the qualitative analysis and the construction of the comprehensive theoretical model; the fifth part is the discussion of the analysis results; the sixth part is the conclusion of the research and management implications.

## 2. Literature

### 2.1. The Connotation Definition of Waste Separation Behavior

Waste separation is a necessary prerequisite for effective waste management, and it is the most effective means to promote the recycling of domestic waste. It is a key link to realize the harmlessness, reduction and resource utilization of waste. Therefore, it is considered as the top priority in domestic waste management (Chung and Poon, 1999) [12]. Based on different perspectives of waste separation, scholars have different definitions of waste separation behavior. Jank et al. (2015) [13] defined waste separation as: Separating the recyclable waste and landfill waste from MSW at the source according to different separation standards, which aims to reduce the difficulty of later disposal, strengthen resource recycling and reduce environmental damage caused by landfill; Areeprasert (2017) [14] believes that the practice of waste source separation is to separate the source of domestic waste in daily life and then transport different types of waste to the waste transfer center for selective material recycling and incineration. Combined with the research of previous scholars, the authors have found that the concepts of waste separation are common in some ways, among which urban residents, sources, classified collection, reutilization and reduction are frequently mentioned words. Moreover, waste separation behavior is defined logically from the process of subject, standard, implementation and goal. In summary, in this study, the authors defined that waste separation behavior refers to that in the process of waste management, urban residents who are the source of waste generation and treatment, collect waste according to specified categories and put it in the designated places, so as to reduce the difficulty of waste disposal and promote the realization of harmless, resource-based and quantified waste.

### 2.2. Structural Analysis of Waste Separation Behavior

Based on the behavioral research, the elaboration of individual behavior selection and behavior manifestation is conducive to systematic and professional research. Furthermore, it can address the problems in behavioral phenomena more effectively. Via referring to the research on the structure of environmental behavior, we found that scholars mainly carry out detailed analysis on environmental behavior based on its manifestation, presentation content, occurrence space, etc. In terms of manifestation, Lee et al. (2013) [15] constructed a structure which includes the seven factors responsible for environmental behavior, namely, civil behavior, financial behavior, physical behavior, persuasion behavior, sustainable behavior, pro-environment behavior, and environment-friendly behavior. In terms of presentation content, Kaiser et al. (2003) [16] classified environmental behaviors according to the content of behaviors, including waste management, water and energy conservation, and resource recovery. In the field of space, we have systematically explored environmental behavior from the home, work and public spheres in our previous research (Chen et al. 2017) [17]. Residents’ waste separation behavior has a certain terminality in content presentation and form of expression, which is difficult to be further refined. However, although the classification based on spatial regions can identify the consistency of individual behaviors in different spatial domains, the specific mechanism characteristics of behaviors is still unclear. In addition, we also found in the previous research that the structural division of behavior from the perspective of behavioral motivation is conducive to understanding the causes of behavior, facilitating the implementation of interventions, and classifying negative undesired environmental behaviors into three categories, that is spontaneous, following and defensive behavior (Chen et al. 2017) [18]. On this basis, in this study, we refined the structure of waste separation behavior from the perspective of behavioral motivation.

### 2.3. Research on Influencing Factors of Waste Separation Behavior

At present, the research on the influencing factors of waste separation behavior mainly focuses on the psychological and situational levels. The psychological level mainly consists of the factors of values and cognition, while the situational level mainly includes the factors of policies, products and facilities. Values are goals or standards that play a guiding role in a person’s life, and they are the main factors that influence the formation of specific attitudes and behaviors (Kristiansen and Zanna, 1994) [19]. Therefore, the discussion on the relationship between values and individual behavioral tendencies has drawn extensive attention. Chen et al. (2014) [20] divided the values into conspicuous consumption values, emotional consumption values, economic consumption values, functional consumption values, and social consumption values in the study of low carbon consumption behavior. Ecological values directly or indirectly affect the formation of individual ecological attitudes (Thompson and Barton, 1994) [21]. Locke (2000) [22] believes that all human behaviors are related to factors such as cognition and emotion. Cognition can play a role in both conscious and unconscious levels, affecting behavioral decision-making (Vassanadumrongdee and Kittipongvises, 2018) [23]. Studies have shown that individuals with more knowledge of waste separation can promote their active participation in waste separation and recycling activities (Echegaray and Hansstein, 2016; Zhang et al. 2017) [24,25]. At the level of situational factors, Barr (2003) [26] divided the situational factors among the influencing factors of urban residents’ energy-saving behavior into policy-based factors and product-based factors. The policy-based factors mainly include policy enforcement and validity. Through examining economic policies and guiding policies, the enforcement and validity of energy conservation policies are measured by using the influence degree of residents. Bernstad (2014) [27] pointed out in his research on household food waste that compared with the guidance of written information, providing facilities and other convenient factors can directly promote residents to separate waste at the source. The above research has made some progress in the field of environmental behavior research, which provides references for this study. However, it is necessary to further analyze the action mechanism of different factors on waste separation behavior. This study will use qualitative analysis to explore the impact of urban residents’ values on their waste separation behavior under the Chinese context.

## 3. Research Methods and Data Sources

### 3.1. Research Methods

The core meaning of waste separation behavior of urban residents is as follows. In the process of waste management, urban residents who are the source of waste generation and treatment classify and collect the waste according to the specified categories and put it in the designated places. The purpose is to reduce the difficulty of waste disposal and promote the realization of harmless, resource-based and quantified waste. In the decision-making process of waste separation behavior, the individuals’ mental state and situational interference will affect the final behavior choice. The purpose of this study is to study the driving mechanism of urban residents’ waste separation behavior. It is necessary to comprehensively analyze the influencing factors of waste separation behavior. The analysis and argumentation of theoretical research are separated from the actual factors and they cannot fully reflect the driving factors in the real situations. Based on this, this study will use exploratory qualitative analysis (quality analysis) to further explore the deep driving factors of urban residents’ waste separation behavior. Furthermore, concepts and variables are linked to form a corresponding theoretical framework and construct a comprehensive theoretical model of the driving mechanism of urban residents’ waste separation behavior.

Qualitative research refers to the research that uses the researcher himself as a research tool, adopting multiple data collection methods in the natural context to carry out the overall study on the social phenomenon. It mainly uses an inductive method to analyze the data and form theory. It is also an activity to obtain the explanatory comprehension of the object’s behavior and significance construction by interacting with the research object (Stebbins, 2006; Charmaz, 2006) [28,29]. It has the function of exploring the social phenomenon, explaining the meaning, and excavating the overall and deep social and cultural structure. The process of qualitative research mainly includes several steps, namely, data collection, theoretical coding and item classification, and theoretical saturation test (Chen et al. 2017; Flick, 2009;) [18,30].

### 3.2. Data Collection

The first step in qualitative research is to collect first-hand information about the respondents. In the interview process of in-depth interviews and questionnaire surveys, this study guided urban residents to participate in the semi-structured interview (survey) outline designed by the authors. From their own point of view, residents expressed their views and attitudes on resource environment and waste separation. Then, they shared what they have seen or heard about waste separation and their experience in daily life. Finally, they focused on their obstacles and driving factors in waste separation in daily life.

Through the communication with students, relatives and friends from different regions before the survey, we knew the gender, age, education background, and working characteristics of the urban residents to be investigated in advance. This aimed to ensure the rationality of sample distribution in gender, age, education background, and working characteristics when conducting stratified sampling. Before the formal interviews and the issuance of the questionnaires, we assured the interviewees that the personal information involved in the survey results would be kept completely confidential and we would give them small gifts to thank them for participating in our research. The interview outline and survey items are shown in Table 1.

### 3.3. Sample Selection

The study was conducted from August 2018 to October 2018. Based on the in-depth interview survey, open questionnaire survey was also adopted to supplement the data obtained from in-depth interviews. In order to ensure the representativeness of the survey samples, this study adopted a pre-stratification method to determine the sample structure to be investigated. Stratified sampling is a method that randomly extracts samples (individuals) from different layers in a specified ratio from a population that can be divided into different subpopulations (or called layers). The sample representativeness of this method is better, and the sampling error is relatively small (Flick, 2009) [30]. According to the different characteristics of China’s geographical distribution and economic structure, three provinces in central, eastern and western China were selected for the research. The urban residents surveyed were distributed in 12 cities in three provinces of China (Jiangsu, Anhui, Gansu). In addition, the specific sample structure is determined based on the actual conditions such as age, education background and work characteristics of China’s urban residents (Table 2).

Jiangsu province, located on the east coast of China, is economically advanced in China. It has a relatively large amount of urban residents’ resource consumption and household waste production. A total of 119 residents from five different cities (from south to north, Suzhou, Wuxi, Nanjing, Yancheng and Xuzhou) were investigated (12 of them were interviewed in depth); Anhui is located in the central and eastern part of China. Its economy is at the middle level of China. The resource consumption and household waste production of urban residents are at the middle level. A total of 107 residents from four different cities (from south to north, Wuhu, Hefei, Huainan and Huaibei) were investigated (ten people were interviewed in depth); Gansu province is located in the western region of China, where the economy is relatively backward and the urban residents’ resource consumption and household waste production are relatively small. A total of 104 residents from three different cities (from south to north, Tianshui, Lanzhou and Jinchang) were investigated (eight of them were in-depth interviews).

In the in-depth interview with 30 urban residents, except for one resident who chose to quit the interview in the middle of the interview (about 17 min), the interview time of each resident was 20–35 min. In addition, an open questionnaire survey was conducted among 300 residents in different cities. As most of the respondents were students, relatives and friends, the survey process was easy to control. At last, a total of 294 valid questionnaires were collected. Finally, the interviewees were recorded, edited and exported with the Audacity Software, and the interview and questionnaire records were sorted out together with written records so as to dig out the deep driving factors of urban residents’ waste separation behavior and help design a more reasonable survey scale.

## 4. Data Analysis and Model Construction

### 4.1. Statistics

A large number of qualitative materials of written content have been formed due to the large number of interviewees (29 in-depth interviews +294 open questionnaires). Therefore, we use the computer-aided qualitative data analysis (CAQDAS) software (Qsr International Pty Ltd, Doncaster, Australia) to analyze the qualitative data and classify or segment the qualitative data containing a large amount of text content for archiving and searching. CAQDAS has the following advantages: (1) Speed up the processing of large-scale data; (2) more accurate in calculating the characteristics of the phenomenon; and (3) promote team research, such as designing consistent coding methods (Kotarba, 1997) [31].

**Figure 1:**
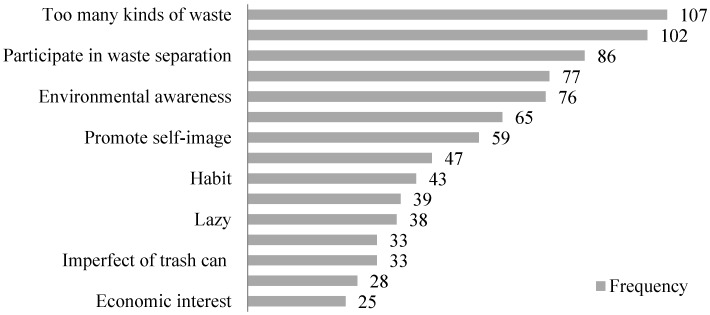
Frequency of key entries in interview data.

When we used CAQDAS to sort out the data, we found that different respondents had the same or very similar answers to the same questions, such as repeated listings, which brought redundancy and complicated obstacles to the research. So the study categorized the same or very similar terms at first. For example, when the interviewees discussed why they were willing (or unwilling) to separate the waste, the word “too many kinds of waste” can be entered into the computer and all these codes can be listed and counted by the CAQDAS program. Two team researchers were invited to sort out and classify the same interview data. Then, they were asked to compare the data with each other to ensure the scientific rigor of the work. The study randomly selected 2/3 sample interviews and surveys (22 in-depth interviews, and 217 open questionnaires) for open coding, axial coding and selective coding analysis. The remaining 1/3 sample interviews and survey records were reserved for theoretical saturation test. The entries with very frequent occurrences are listed in Figure 1.

According to the statistical analysis, 107 out of 239 respondents mentioned the phrase "too many kinds of waste" which accounted for the highest proportion. What is more, 102 out of 239 respondents mentioned the word “unclear separation standards”, which ranked second. From what has been discussed, it can be seen that the interviewees have a high consistency in the cognition of waste problem. In addition, "participation in waste separation", "trouble", "environmental awareness", “wasting time”, “promoting self-image”, “improving living environment”, “habit”, “incomplete facilities”, “laziness”, “good for health”, “imperfect trash can”, “imperfect policy and laws”, and “economic interest” were mentioned more than 20 times. This work laid a good basis for the subsequent coding work.

Coding in qualitative research is a program for analyzing data, including asking questions about text and constantly comparing phenomena, concepts, etc. However, classification summarizes these concepts into superior concepts and the relationship between categories and superior categories [30]. The methods of coding and classification mainly include theoretical coding, open coding, axial coding, selective coding, qualitative content analysis, etc. (Wakeford, 2012) [32]. Coding and classification methods are usually used together. Open encoding, axial encoding and selective encoding are the most classical combination methods in analyzing text data, which can accurately compress and simplify the data and then classify related concepts. In this study, open coding, axial coding and selective coding are used together to encode and classify the collected data.

### 4.2. Open Coding

After the interview, via the initial collation of the data, the open coding was carried out in the first step. Some concepts were sorted out in the first interview, then the correlation and difference between these concepts were analyzed, and some categories were summarized. The second interview was conducted according to the problems found in the coding process and the concept categories sorted out. This cycle was repeated until the coder thought the concepts and categories of coding were relatively rich. Relevant concepts and categories were repeated in the coding process, so that the interview can no longer continue and the coding can proceed to the next level. Table 3 and Table 4 reflect the process of conceptualization and categorization of the original interview records in this paper, and the results of categorization are the relevant influencing factors of urban residents’ waste separation behavior. Considering the space limitation, in this paper, we only selected representative original record statements and initial concepts for each category.

### 4.3. Axial Coding

The second-level coding is the spindle coding which is also known as associative registration or axis registration. Its main task is to discover potential logical connections between categories. During the axial coding, the researcher only conducted an in-depth analysis of one category at a time. Then, the researcher further explored related relationships centering on this category and analyzed whether each category has a potential correlation at the conceptual level. Thus, it is called “axis” or “spindle”. After analyzing the correlation between each group of categories, it is also necessary to identify the level of the category within the group, that is, to identify the main category and sub-categories, and then establish the relationship between the main category and the sub-categories under continuous comparative analysis. The formation process of the main category (axial coding process) is shown in Table 5.

### 4.4. Selective Coding

Selective coding is also known as core login or selective login. It excavates the core category from the main category, and then analyzes the connection between the core category and other categories. It describes the behavioral phenomena and context conditions in the way of a “story line” and develops a new theoretical framework on this basis. The typical relationships of the main categories of this study are shown in Table 6.

This study identified the core category of “the driving mechanism of waste separation behavior”. The “story line” around the core category can be summarized as: The three main categories, namely, separation empowerment perception, individual psychology factors and situational factors are the internal driving factors of waste separation behavior, which have a significant influence on waste separation behavior and directly determine the public’s waste separation behavior. Meanwhile, separation empowerment perception and situational factors can be used as moderating variables to regulate the connection between individual psychology factors-waste separation behavior and separation empowerment perception-waste separation behavior. Based on this "story line", in this study, we constructed and developed a new driving mechanism framework for waste separation.

### 4.5. Saturation Test

Edgington (1967) [33] proposed the criterion of "theoretical saturation" which is used to judge whether the sampling of a certain structural category is saturated or not. In this case, saturation means that it is no longer possible to find additional data so that scholars can develop more features of this category. In this study, eight additional interviews and 77 open questionnaires (about 1/3 of the total samples) were used for the theoretical saturation test. The results show that the types and categories in the model have been developed in a very rich way, and no new important categories or relationships and new constituent factors have been found. Therefore, we can conclude that the structure of the above waste separation behavior and its driving factors is theoretically saturated.

### 4.6. Model Establishment

**Figure 2:**
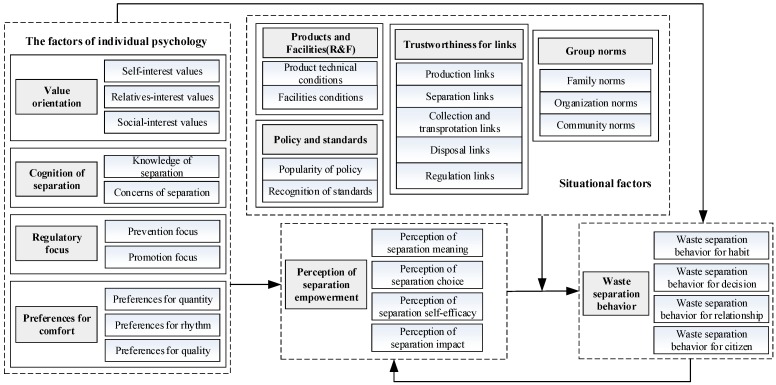
Driving model of urban residents’ waste separation behavior.

Based on qualitative research, the separation behavior of urban residents and its driving factors were explored, and the core research variables were defined. Further, a driving theoretical model for urban residents’ waste separation behavior and a model of urban residents’ response to the guiding policy were constructed. Then, relevant research hypotheses were proposed based on the qualitative analysis results and the theoretical and documentary research. A comprehensive research model (Figure 2) was constructed from the perspective of behavior driving, policy response and policy simulation.

## 5. Discussion

This study defined the waste separation behavior of urban residents. According to the qualitative analysis, it also determined the structural dimensions of waste separation behavior, namely, waste separation behavior for habit, waste separation behavior for decision, waste separation behavior for relationship, and waste separation behavior for citizen.

As for the study of the dimensions of environmental behavioral structures, few studies classify such behavior from the perspective of motivation. Chen et al. (2017) [18] divided undesired environmental behaviors into three types: spontaneous, following and defensive from the perspective of motivation. Human behaviors are determined by various factors when combined with the research on behavior selection. Chen et al. (2014) [34] believe that individual environment and low-carbon behaviors are correlated with their environmental knowledge, and individuals with low environmental knowledge are more likely to damage the environment and engage in behaviors of high-carbon consumption. In addition, values can influence individual behaviors through mechanisms such as habits (Courbalay et al. 2015) [35]. Cooke (2015) [36] found that there was a significant correlation between individual environmental values and environmental behaviors. Therefore, firstly, this study speculated that individual knowledge, values and other factors would affect their habitually occurring waste separation behavior, which we call "waste separation behavior for habit". Secondly, in order to ensure the maximization of self-interest, individuals tend to choose the same behavioral strategies as others in the absence of information (Jarkko and Emilia, 2015) [37]. With regards to waste separation behavior, individuals will separate the waste actively or passively due to a sense of interest, such as economy, health and so on, which we call "waste separation behavior for decision". Thirdly, individuals have the attribute of convergence. Considering self-protection and interpersonal estrangement, individuals will choose to ignore certain behaviors and opinions so as to maintain consistency with the group (Deniz et al. 2013) [38]. Therefore, if an individual chooses to separate waste due to defensive purposes when he needs to make a choice between separation behavior and interpersonal relationship, we call it "waste separation behavior for relationship". In addition, there is a strong correlation between moral responsibility and environmental behavior (Stern, 2000) [39]. This sense of responsibility encourages residents to foster the will to protect the ecology and benefit the society, and then carry out waste separation, which we call "waste separation behavior for citizen ".

The study found that the factors of individual psychology can affect the waste separation behavior of urban residents, including value orientation, cognition of separation, regulatory focus and preferences for comfort. Individual values are the decisive factors of behavior, especially the individual ecological values will directly or indirectly affect their environmental attitudes and behaviors. Through qualitative analysis, we found that individuals with different values tend to have inconsistencies in separation, and there are differences in the views of self-interested and relative-interested individuals on waste separation. This study divided the subject of a person’s values for profit into the individual’s own layer, the relationship layer and the social layer according to the circle. Then, these three layers were extended to self-interest values, relative-interest values and social-interest values. Separation cognition is the individual mastery of the knowledge of waste separation and the amount of attention paid to the related information of waste separation. According to Chen et al.’s (2017) [40] explanation of the connotation of environmental cognition, this study divided the separation cognition into two aspects: separation knowledge and separation concerns. Regulatory focus includes prevention focus and promotion focus, which expresses that the individual’s difference in self-regulation of the psychological feedback mechanism would result in his/her different attention and choice of external stimulus, thus affecting his/her behavioral choice. During the interviews, the authors found that different individuals focused on different things, some focused on the positive impact of separation, while others were more sensitive to the negative effects such as health damage caused by non-separation. Residents’ comfort requirements and preferences for their own life would affect their environmental behavior. Through interviews, it is found that preferences for psychological comfort of urban residents are mainly reflected in preferences for quantity (the pursuit of scale, scene, etc.), preference for rhythm (the pursuit of time urgency and efficiency), and preference for quality (the pursuit of the quality of life). It is found that separation empowerment perception affects the waste separation behavior of urban residents. The theory of Involvement points out that individuals’ perceptions of participation in events will increase their investment degree (Milem and Berger, 1997) [41]. Since then, scholars have expounded this phenomenon with the concept of psychological empowerment to express the individual’s psychological perception of being endowed with rights. Psychological empowerment has been widely applied in the field of organizational management. It is found that different dimensions of psychological empowerment have different impacts on different organizational citizenship behaviors. Self-efficacy will promote individual organizational responsibility and interpersonal relationship (Ginsburg et al. 2016) [42]. Similarly, in the process of the source separation of waste, individuals can also be endowed with such psychological rights to enhance their sense of self-efficacy, environmental significance, etc., and strengthen their internal environmental motivation. In this situation, individuals form perceptions of separation meaning through judging the values and significances of waste separation behavior by their own values and standards. They also form the perception of separation self-efficacy by judging their own ability to correctly carry out waste separation and recycling. At the same time, perception of separation choice also enhances the autonomy and dominance of separation, which increases the sense of involvement of residents in separation. In addition, the individuals’ perception of the influence degree of their waste separation behavior, that is, perception of separation impact, will further promote or inhibit their future separation behavior.

It is found that situational factors can affect the waste separation behavior of urban residents from the following aspects: (1) Products and facilities: The reason why most people are not willing to do waste separation is their denial of the products of separation (the health and scalable practicality of recycled products) and the technology (the compatibility of infrastructure and the completeness of the entire recycling system). Based on this, this study divided the factors of products and facilities into products’ technical conditions and facilities’ conditions. (2) Policy and standards: The popularity of urban residents’ waste control policies mainly consists of four aspects: the popularity of technical standards, the popularity of fees, the popularity of command and control policies, and the popularity of participation in regulation. It is mainly used to examine the effect of the existing relevant waste control policies and to measure the intensity and perfection of the policies. The recognition of standards is because the separation of waste needs a standard to rely on. The operability of standard rules and the distinguishability of waste categories are the basis of waste separation of residents by following the reference scale. They also determine the difficulty and willingness of urban residents in waste separation. (3) Link trustworthiness: In the whole process of waste management, there are multiple subjects involved in production, separation, collection, transportation, treatment, regulation and other links, and weaknesses or deficiencies in each link will cause important negative impacts on other links. According to the theory of trust, optimistic psychological expectations and acceptable risk willingness are important conditions for forming mutual trust in each link which can avoid behavioral deviations and achieve goals. (4) Group norms: When facing environmental problems, what the public feel, such as the atmosphere of community public opinion, the value orientation of group, the criterion of moral evaluation and so on, all belong to the category of group norms. At the same time, the role of residents will change with space. In the process of different roles and space transformation, the role expectation of individuals and the connotation of group norms will also change. Combined with our previous research on environmental behavior, we divided the individual activity space into a home area, work area and public area (Chen et al. 2018) [40]. Based on this spatial division method and the results of qualitative analysis, this study defined the group norms that urban residents are exposed to as three categories: family norms, organization norms and community norms.

Based on our previous studies on environmental behavior dimensions and its influencing factors, this study analyzed the influencing factors and action paths of urban residents’ waste separation behavior through qualitative research and comprehensively constructed the driving model of urban residents’ waste separation behavior. Qualitative research is a qualitative research method. Although it can analyze the category characteristics of related variables and present the corresponding story relationships, the relationship between variables lacks a quantitative empirical test. In future research, it is possible to quantify various factors affecting waste separation behavior of urban residents by means of questionnaires, regression analysis, experimental methods, etc., and to correct the driving model of urban residents’ waste separation behavior.

## 6. Conclusions

Based on the logical process of the main body, standard, execution, and target of the waste separation activity of residents, in this study, waste separation behavior is defined as that in the process of waste management, urban residents, as the source of waste generation and treatment, separate and collect the waste according to the specified categories and put them in the designated places, so as to reduce the difficulty of waste disposal and promote the realization of harmless, resource-based and quantified waste. Furthermore, from the perspective of behavioral motivation, this paper constructed and verified the four-dimensional structure of waste separation behavior, including waste separation behavior for habit, waste separation behavior for decision, waste separation behavior for relationship, and waste separation behavior for citizen. A qualitative research method was adopted to sort out the in-depth interview data of 323 residents of representative cities in the eastern, central and western parts of China. Then, open coding, axial coding and selective coding were carried out. We clarified the main driving factors of urban waste separation behavior and its mechanism of action and built the theoretical model of the driving mechanism of urban residents’ waste separation behavior in 10 main categories such as value orientation, cognition of separation, regulatory focus, preferences for comfort, separation empowerment perception, policy and standards, group norms, link trustworthiness, and social demography variables; and four types of typical relationship structures are proposed, which are: (1) The factors of individual psychology, perception of separation empowerment and situational factors directly affect waste separation behavior; (2) the factors of individual psychology act on waste separation behavior through separation empowerment perception; (3)situational factors have a moderating effect on the influence of separation empowerment perception on waste separation behavior; and (4) factors of individual psychology can react to their separation empowerment perception.

In addition, qualitative analysis also found and defined the connotation and structure of the relevant main categories: (1) The four-dimensional structure of separation empowerment perception is the perception of separation meaning, perception of separation choice, perception of separation self-efficacy, and perception of separation impact; (2) the three-dimensional structure of value orientation, namely, self-interest values, relative-interest values, and social-interest values; (3) cognition of separation includes two dimensions of knowledge of separation and concerns of separation; (4) preferences for comfort are mainly reflected in three aspects: preferences for quantity, preferences for rhythm and preferences for quality; (5) products and facilities include products’ technical conditions and facilities’ conditions; (6) policy and standards include two dimensions of popularity of policy and regulation of standards; (7) group norms are mainly reflected in three aspects: family norms, organization norms and community norms; (8) the five-dimensional structure of link trustworthiness includes production links, separation links, collection and transportation links, disposal links, and regulation links.

**Figure 3:**
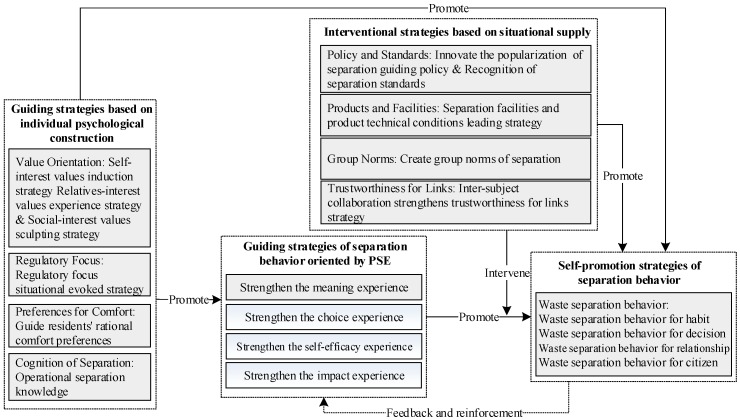
Frame of driving recommendation of waste separation behavior.

To improve the effectiveness of government policies and effectively guide urban residents to carry out waste separation, the study further explored the responding mechanism of urban residents to the guiding policy of waste separation behavior (Figure 3), so as to grasp the practical basis and practical guarantee of guiding policy.

(1) Guiding strategies based on individual psychological construction. From the aspect of value orientation, the government should implement strategies of the induction of self-interest values, the experience of relatives’ interest values, and the shaping of social-interest values. Specifically, it is necessary to encourage residents to recognize that the separation of waste is a self-interested activity, strengthen the value experience of waste separation for the residents’ relationship groups, and arouse the residents’ environmental citizenship awareness. In terms of regulatory focus, it is necessary to implement the situational induction strategy of regulatory focus. Regulatory focus can be induced by short-term situations, the positive motivation state of individual regulatory focus can start, and the sensitivity of residents to waste separation can be improved through the specific design of the background and content in government policy publicity and related education. In terms of preferences for comfort, residents should be guided to rationally pursue life comfort, and the direct and indirect waste of resources should be reduced as much as possible while meeting the comfortable requirements of residents in terms of life time, face and quality. In terms of the cognition of separation, in order to strengthen the operational separation knowledge, the government should spread a clear, specific and operable environmental protection behavior guide to the public and avoid the occurrence of an “over-limit effect” caused by too many abstract communication slogans.

(2) Interventional strategies based on situational supply. In terms of policy and standards, it is necessary to innovate the method of separation to guide the popularity of policy, so as to promote the recognition of waste separation standards. Standards developers can refine the operation standards and specify how the corresponding waste should be delivered. In terms of products and facilities, government should implement the look-ahead strategies of the facilities of separation and products technical conditions. At the present stage, it is urgent to adopt an environmental protection plan of “facility first” and improve the urban spatial layout and planning system, collection, transportation, supply and demand system, and recycling system of waste products. In terms of group norms, the government should actively create group norms of separation. The government can reduce the negative influence of social pressure atmosphere and promote the formation of a positive social pressure atmosphere by spreading education, social marketing and economic incentives. At the same time, policy makers need to establish a guiding mechanism to eliminate individual responsibility decentralization, so as to help residents clarify their role in the process of ecological environment construction and avoid the dispersion of responsibility and free-riding phenomenon caused by group attributes. As for link trustworthiness, the strategy of strengthening the link trustworthiness through inter-agency cooperation should be implemented, and a complete policy system is established in five links of production, separation, collection and transportation, terminal treatment, and regulation.

(3) Guiding strategies of separation behavior oriented by the perception of separation empowerment. The government should strengthen the perception of separation meaning, perception of separation choice, perception of separation self-efficacy, and perception of separation impact. Managers need to inculcate the sense of separation into residents and enhance their awareness of the danger of waste pollution and waste; to strengthen residents’ perception of separation choice (autonomy, sense of control) and positively promote their waste separation; strengthen residents’ own separation literacy, promote their confidence in waste separation and their perception of own ability; and create a benign waste separation atmosphere and increase the social expectations and identity of separation.

(4) Self-promotion strategies of separation behavior. (1) The development strategy of waste separation behavior for habit. The government should attach importance to the cultivation of an individual environmental protection concept in families, realizing the family’s integration of children’s environmental protection cognition and environmental protection behavior education, so as to realize the cultivation of residents’ separation behavior in family life. (2) The inductive strategy of waste separation behavior for a decision. On the one hand, the government can magnify the benefits of waste separation through economic incentives and other means. On the other hand, it should reduce the costs of separation and reduce the factors which make residents resistant to waste separation due to time and economy. (3) The driving strategy of waste separation behavior for a relationship. The following defensive motivation caused by interpersonal relationship is one of the main reasons for individual separation behavior. Therefore, the government should start from enhancing social expectation and social benefits to carry out targeted interventions to residents’ waste separation behavior. (4) The incentive strategy of waste separation behavior for a citizen. Waste separation behavior for a citizen refers to the fact that the residents’ waste separation behavior is out of the sense of responsibility for society and civil consciousness. Therefore, the government can stimulate the consciousness of social responsibility of the residents in the following three aspects. The first is to strengthen education, the second is to improve the system of laws and regulations, and the third is to carry out the practical activities so as to form a broad consensus on waste separation in the whole society.

## Figures and Tables

**Table 1:** Interview outlines and survey items.

Interview Theme	Main Content Outline
Waste separation behavior refers to that in the process of waste management, urban residents, as the source of waste generation and treatment, separate and collect waste according to specified categories and put them in designated places, so as to reduce the difficulty of waste disposal and promote the realization of harmless, resource-based and quantified waste.	
Basic Information	gender, age, income level, education, occupation, family structure, city
Related cognition and driving factors of waste separation behavior	What do you think about the current resource and environment problems? What’s your opinion on waste separation? Why do you (not) need waste separation? How do you practice waste separation in your daily life? Why are you and others around you (not) willing to do waste separation? What is your starting point (not) for waste separation?

**Table 2:** Sample structure.

Social Demographic Variables		Frequency (Interviewed in Depth + Open Questionnaire Survey)	Percentage	Social Demographic Variables		Frequency (Interviewed in Depth + Open Questionnaire Survey)	Percentage
Gender	Male	15 + 152	51.70%	City	Suzhou	3 + 26	8.98%
	Female	14 + 142	48.30%		Wuxi	2 + 15	5.26%
Education background	Junior high school or below	4 + 42	14.24%		Nanjing	3 + 27	9.29%
	Senior high school or technical secondary school	5 + 63	21.05%		Yancheng	2 + 15	5.26%
	Junior college	6 + 77	25.70%		Xuzhou	2 + 20	6.81%
	Bachelor	9 + 69	24.15%		Wuhu	3 + 26	8.98%
	Master or above	5 + 43	14.86%		Hefei	3 + 32	10.84%
Age	≤20	3 + 31	10.53%		Huainan	2 + 19	6.50%
	21–30	5 + 52	17.65%		Huaibei	2 + 20	6.81%
	31–40	8 + 76	26.01%		Tianshui	2 + 33	10.84%
	41–50	7 + 69	23.53%		Lanzhou	3 + 35	11.76%
	51–60	4 + 43	14.55%		Jinchang	2 + 26	8.67%
	>60	2 + 23	7.74%				

**Table 3:** Process and results of open coding for waste separation behavior.

Source Statement (Representative Statement)	Category
Usually, there is no habit of recycling and separating, and the waste is directly thrown into the trash can. We don’t care which side is recyclable and which side is not.	Waste separation behavior for habit
I separate waste to see if it has value. I will choose to separate in the case of bottles and cartons which can be sold.	Waste separation behavior for decision
In public, I will not litter but also more willing to separate waste.	Waste separation behavior for relationship
As a social citizen, I feel it is necessary for me to contribute to the society and green ecology	Waste separation behavior for citizen

**Table 4:** Process and results of open coding for the driving factors of waste separation behavior.

Source Statement (Representative Statement)	Category
The publicity of the separation policy is not enough, and many of the policies are not clear.	Popularity of policy
The standard of waste separation is not clear and is not well understood.	Regulation of standard
China’s waste separation and recycling technology is still relatively deficient at present.	Product technical conditions
There is only one trash can downstairs in the community which is not marked as recyclable and non-recyclable, so we are powerless.	Facilities conditions
I think family norms have a great influence on personal waste separation behavior.	Family norms
I think there should be organization norms first. I pay more attention to the behavior of my colleagues.	Organization norms
Now the whole community has no atmosphere of separation, and I can’t change anything by myself.	Community norms
Now the recyclable packaging products should not be very popular, and the price factor makes the public not use recyclable packaging products.	Production links
Most people do not attach importance to waste separation and recycling, and many laws are not universal.	Separation links
When the waste is collected and transported, the waste will be mixed and the waste collection efficiency is not high.	Collection and transportation links
Now the way to deal with waste is either landfill or incineration, so waste separation is meaningless.	Disposal links
The regulation and punishment are insufficient and the regulator does not attach importance to waste separation.	Regulation links
If the waste is not separated, it will pose a great threat to my health.	Self-interest values
For the health of my family and friends, I am willing to do waste separation.	Relative-interest values
Waste separation is the responsibility that should be fulfilled as a citizen.	Social-interest values
I know some relevant knowledge about waste separation. For example, I know that waste in our country is mainly divided into four categories: recyclable waste, kitchen waste, toxic and harmful waste, and other waste.	Knowledge of separation
I pay close attention to the news about the waste separation policies or the introduction, revision and other aspects of standards.	Concern of separation
A lot of waste will cause great pollution to the environment. For example, discarded batteries contain toxic substances such as metallic mercury and cadmium, which will cause serious harm to human beings.	Prevention focus
Waste separation can save resources, and a lot of waste can be reused after recycling, which can greatly alleviate the pressure brought by scarce resources.	Promotion focus
I believe in saying "more is better", of course, the more the better.	Preferences for quantity
I like fast-paced life, sometimes I feel at loose ends when I am idle.	Preferences for rhythm
I am very particular about life quality and I never compromise.	Preferences for quality
I think it is very important to separate household waste, which is good for the country and people.	Perception of separation meaning
I have the right to decide whether to report to the relevant departments for those who dispose waste at will. I don’t want to be told or pressured to do so.	Perception of separation choice
I have mastered a lot of knowledge about waste separation, and I know how to separate waste effectively.	Perception of separation self-efficacy
I feel that I have the ability and confidence to persuade people around me to actively participate in waste separation.	Perception of separation impact

**Table 5:** Process and results of axial coding.

The Connotation of Category Relations	Corresponding Subcategories	The Main Categories
Urban residents carry out waste separation as a behavioral activity out of daily habits.	Waste separation behavior for habit	Waste separation behavior
The waste separation by urban residents is the result of the decision after weighing the interests of economy and health.	Waste separation behavior for decision	
Waste separation by urban residents is a behavioral choice influenced by others’ behaviors and attitudes.	Waste separation behavior for relationship	
Urban residents carry out waste separation as a behavioral activity out of civic consciousness and to build a better home.	Waste separation behavior for citizen	
The popularity of policy is a policy-level situational factor that affects the separation of household waste.	Popularity of policy	Policy and standards
The regulation degree of the standards is a policy-level situational factor that affects the separation of household waste.	Regulation of standard	
The products that sale on the market and the production techniques are the situational factors of product facilities that affect the separation of household waste.	Product technical conditions	Products and facilities
The condition of existing infrastructure construction is the situational factor of product facilities that affect the separation of household waste.	Facilities conditions	
Family members’ views on waste separation are group normative situational factors that affect the separation of household waste.	Family norms	Group norms
The views of colleagues on waste separation are group normative situational factors that affect the separation of household waste.	Organization norms	
The views of community and other people around on waste separation are group normative situational factors that affect the separation of household waste.	Community norms	
The behavioral trust of the main part of the production process in the waste management process is a group normative situational factor that affects the separation of household waste.	Production links	Link trustworthiness
The behavioral trust of other subjects in the separation process in the waste management process is a group normative situational factor that affects the separation of household waste.	Separation links	
The behavioral trust of the main body in the process of waste collection and transportation is a group normative situational factor that affects the separation of household waste.	Collection and transportation links	
The behavioral trust of the subjects in the process of waste management is a group normative situational factor that affects the separation of household waste.	Disposal links	
The behavior trust of the main body in the regulation link in the process of waste management is a group normative situational factor that affects the separation of household waste.	Regulation links	
The self-interest values of urban residents are the value factors of individual psychological level that affect the separation behavior of waste.	Self-interest values	Value orientation
The relative interest values are the value factors of the individual psychological level that affect the waste separation behavior.	Relative-interest values	
The social interest values of urban residents are the value factors of the individual psychological level that affect the waste separation behavior.	Social-interest Values	
The knowledge of separation of urban residents is the cognitive factor of the individual psychological level that affects the waste separation behavior.	Knowledge of separation	Cognition of separation
The concern of separation of urban residents is the cognitive factor of the individual psychological level that affects waste separation behavior.	Concern of separation	
The prevention focus of urban residents is the individual psychological level factor that affects waste separation behavior.	Prevention focus	Regulatory focus
The promotional focus of urban residents is the individual psychological level factor that affects the waste separation behavior.	Promotion focus	
The preferences for quantity of urban residents are the individual psychological level factors that affect waste separation behavior.	Preferences for quantity	Preferences for comfort
The preferences for rhythm of urban residents are the individual psychological level factors that affect waste separation behavior.	Preferences for rhythm	
The preferences for quality of urban residents are the individual psychological level factors that affect waste separation behavior.	Preferences for quality	
Individual perception of separation meaning will further affect the occurrence of waste separation.	Perception of separation meaning	Perception of separation empowerment
Individual perception of autonomy brought by their waste separation behavior will further affect the occurrence of their waste separation behavior.	Perception of separation choice	
Individual perception of the efficacy of waste separation behavior will further affect the occurrence of waste separation.	Perception of separation self-efficacy	
Individual perception of separation impact will further influence the occurrence of their waste separation behavior.	Perception of separation impact	

**Table 6:** Results of selective coding.

The Connotation of Relational Structure	Typical Relational Structure	Core Category
Separation empowerment perception is the internal driving factor of waste separation behavior, which directly determines whether an individual will conduct waste separation behavior or not.	Separation empowerment perception→ Waste separation behavior	The driving mechanism of waste separation behavior
Waste separation behavior can strengthen separation empowerment perception and bring about a positive experience of separation empowerment.	Waste separation behavior→Separation empowerment perception	
The individual psychological factors are the internal driving factors of waste separation behavior, which directly determine whether they will conduct waste separation behavior or not.	The individual psychology factors→ Waste separation behavior	
Situational factors are the internal driving factors of waste separation behavior, which directly determine whether they will conduct waste separation behavior or not.	Situational factors→ Waste separation behavior	
Value orientation, cognition of separation, regulatory focus, and preferences for comfort will determine the individual perception of a certain behavioral result. In other words, whether the behavior can give satisfaction to their perception of separation meaning, perception of separation choice, perception of separation self-efficacy, perception of separation impact, and other aspects directly influences them to conduct waste separation behavior or not.	The factors of individual psychology →Separation empowerment perception→ Waste separation behavior	
Policy and standards, products and facilities, group norms, and link trustworthiness and other situational factors are external constraints of waste separation behavior. As moderating variables, situational factors affect the relational strength and direction between separation empowerment perception and waste separation behavior.	Situational factors→ Separation empowerment perception→ Waste separation behavior	
